# Mechanisms of Ion Transport Across the Mouse Retinal Pigment Epithelium Measured In Vitro

**DOI:** 10.1167/iovs.61.6.31

**Published:** 2020-06-15

**Authors:** Sunna Bjorg Skarphedinsdottir, Thor Eysteinsson, Sighvatur Sævar Árnason

**Affiliations:** Department of Physiology, BioMedical Center, Faculty of Medicine, University of Iceland, Reykjavik, Iceland

**Keywords:** mouse, retinal pigment epithelium, ion transport, Ussing, NaK-ATPase

## Abstract

**Purpose:**

To examine ion transport across the mouse retinal pigment epithelium (RPE), measured by the short-circuit current (I_SC_) and transepithelial resistance (TER).

**Methods:**

Sheets of RPE from mice (C57BL6/J) with retina, choroid, and sclera attached were mounted in Ussing chambers (0.031-cm^2^ aperture) and Krebs solution. The I_SC_ and TER were recorded with voltage clamps. Receptors implicated in ion transport were blocked or stimulated by ligands applied to both sides.

**Results:**

The mean initial I_SC_ was −12.0 ± 3.9 µA/cm^2^ (basolateral negative), and mean TER was 67.1 ± 8.0 ohm·cm^2^. RPE preparations remained stable for 3 hours, with I_SC_ decreasing by 0.078 ± 0,033 µA/cm^2^/hr. Adenosine triphosphate (100 µM) increased I_SC_ by 2.22 ± 0.41 µA/cm^2^ (P = 0.003). Epinephrine (100 µM) increased I_SC_ by 1.14 ± 0.19 µA/cm^2^ (*P* = 0.011). Bumetanide (100 µM) reduced I_SC_ by 1.72 ± 0.73 µA/cm^2^ (*P* = 0.027). Ouabain (1 mM) induced a biphasic response: an I_SC_ increase from −7.9 ± 2.4 to −15.49 ± 2.12 µA/cm^2^ and then a decrease to −3.7 ± 2.2 µA/cm^2^. Ouabain increased TER by 15.3 ± 4.8 ohm·cm^2^. These compounds were added sequentially. Apical [K^+^]_o_ at zero mM transiently increased I_SC_ by 3.36 ± 1.06 µA/cm^2^. Ba^++^ decreased I_SC_ from −10.4 ± 3.1 to −6.6 ± 1.8 µA/cm^2^ (*P* = 0.01). Ba^++^ reversed the K^+^-free response, with I_sc_ decreasing further from −5.65 ± 1.24 to −3.37 ± 0.79 µA/cm^2^ (*P* = 0.029).

**Conclusions:**

The I_SC_ and TER can be recorded from the mouse RPE for 3 hours. Adrenergic and purinergic receptors affect murine RPE ion transport. Sodium–potassium adenosine triphosphatase plays a role in net ion transport across mouse RPE, and Na-K-2Cl cotransporter activity partly accounts for transepithelial ion transport. Mimicking light-induced changes, low subretinal [K^+^]_o_ increases ion transport transiently, dependent on K^+^ channels.

The retinal pigment epithelium (RPE) is a monolayer of pigmented cells that lies between the sclera and the neural retina in the eye. It forms a part of the blood–retinal barrier and serves to adjust the ionic environment around the outer segments of the photoreceptors in response to light and darkness.^1^^–^^3^ Many of the transport mechanisms of fluid and ions across the RPE are known and have been demonstrated in several species.^4^^–^^6^ The RPE has been characterized as a fluid-absorbing epithelium, based on the polarity of Na-K-2Cl cotransporters and Cl channels.^7^^–^^9^ In fluid-absorbing epithelia, the cotransporters are located on the apical cell membrane and the Cl channels on the basolateral membrane, and this appears to be the case with the RPE.^4^^,^^6^^,^^10^^,^^11^ It has been known for some time that there is a transepithelial potential (TEP) difference across the RPE of up to 30 mV, depending on the species, with the retinal (apical) side positive with respect to the choroidal (basolateral) side.^12^^,^^13^ The ion current that accounts for the potential difference is mediated partly by transport of ions such as potassium from the retina to the choroid and partly by the transport of ions such as sodium and chloride from the choroid to the retinal side.^3^^,^^13^^–^^15^ The net transepithelial ion current can be measured electrophysiologically if the TEP is voltage clamped to zero (i.e., under short-circuit conditions using the Ussing technique).^16^

A great deal of information has been gathered about the mechanisms involved in transepithelial transport across the RPE in a variety of vertebrate species by utilizing the Ussing technique, which is commonly used to evaluate changes in ion transport across epithelia.^5^^,^^14^^,^^15^^,^^17^^–^^19^ However, a notable exception is the mouse RPE, with only one attempt reported in the literature.^20^ This is surprising, as murine models of RPE dysfunction have been developed in recent years that may involve abnormal fluid and ion transport in some cases.^2^^,^^21^^–^^23^ The small size of mouse eyes, however, has hampered their use in studies involving standard electrophysiological techniques such as the Ussing chamber. A successful procedure for recording the short-circuit current (I_SC_) and transepithelial resistance (TER) reliably from the murine RPE would provide an opportunity to examine ion transport in both healthy mice and those with mutations affecting the RPE. In this study, we have developed a procedure that allows for measuring the I_SC_ and TER from mouse pigment epithelium attached to the neuroretina and the choroid, as well as with the sclera in situ. We have tested the viability of the preparation and evaluated the function of some of the transport mechanisms in the mouse that are known to occur in the RPE of a variety of other species.

## Methods

### Experimental Animals and Tissue Preparation

**Figure 1.**
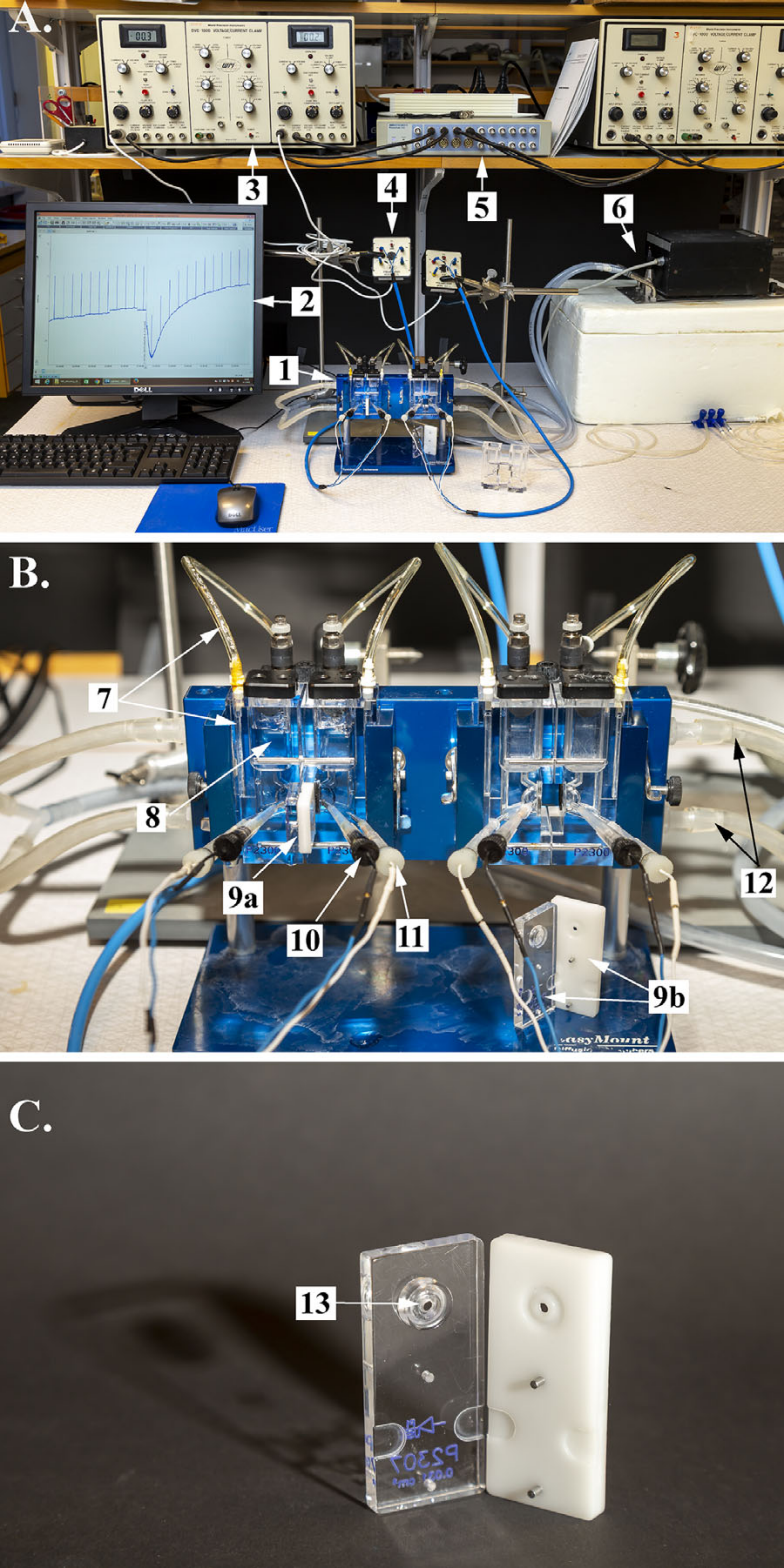
Photographs of the experimental setup providing an overall view of the setup, with the components indicated by *arrows* and adjacent numbers as follows: (**A**) 1. Ussing chambers; 2. LabChart data acquisition software; 3. voltage-clamp unit; 4. preamplifier; 5. analog/digital converter; and 6. thermocirculator. (**B**) 7. Air tubes; 8. bath with Krebs solution; 9a. insert with tissue in place; 9b. open insert; 10. voltage electrodes; 11. current electrodes; and 12. thermocirculator inflow tubes. (**C**) 13. Aperture on insert.

Healthy adult mice (C57Bl/6J), purchased from Taconic Denmark (Silkeborg, Denmark) were kept on a regular 12:12 light/dark cycle. All animal experiments were approved by the Icelandic Food and Veterinary Authority (MAST license numbers 0112-0101 and 0312-0102), and all experimental procedures adhered to the ARVO Statement for the Use of Animals in Ophthalmic and Vision Research. The animals were sacrificed at about the same time in the morning, as it is known that circadian rhythms and light/dark entrainment can affect the RPE short-circuit current.^24^ One animal at a time was anesthetized with carbon dioxide (CO_2_) in a closed box for 15 to 20 seconds; thereafter, a cervical dislocation was performed, and the carotid arteries were severed. Whiskers were removed, and the eyes were enucleated and placed in a Petri dish with a cold Krebs solution. Using a 0.45 × 12-mm Sterican needle (B. Braun, Melsungen, Germany), a small puncture was then made in the cornea of one eye under a microscope, and the cornea and the iris were removed with incisions along the border of the pars plana. The lens was gently removed by pressure to avoid retinal detachment. Details of the Ussing chamber recording system are shown in Figure 1. The RPE, together with the retina, choroid and sclera, was mounted in miniature epithelial chambers with an aperture of 0.031 cm^2^ (EasyMount; Physiologic Instruments Inc., San Diego, CA, USA), depicted in Figures 1B and 1C, with a normal Krebs solution on both sides kept at 38°C by a thermocirculator (Heto Lab Equipment, Allerod, Denmark) and aerated with an air mixture of 5% CO_2_ and 95% O_2_.

### Drugs and Solutions

Adenosine triphosphate (ATP) was purchased from Tocris Bioscience (Oxford, UK), and epinephrine, bumetanide, and ouabain were purchased from Sigma-Aldrich (St. Louis, MO, USA). The normal Krebs solution had the following final bath ion concentration (mmol/L): Na^+^, 138.6; K^+^, 4.6; Ca^2+^, 2.5; Mg^2+^, 1.2; Cl^–^, 125.1; HCO_3_^–^, 21.9; PO4^2^^–^, 1.2; SO4^2^^–^, 1.2; and glucose, 11.1. A K^+^-free Krebs solution was made by replacing K^+^ ions with Na^+^, with the following composition (mmol/L): Na^+^, 143.2; K^+^, 0.0; Ca^2+^, 2.5; Mg^2+^, 1.2; Cl^–^, 125.1; HCO_3_^–^, 21.9; PO_4_^2^^–^, 1.2; SO_4_^2^^–^, 1.2; and glucose, 11.1. The osmolarity of these two types of Krebs solutions was the same and measured to be 295 ± 5 mOsm. All solutions were aerated with an air mixture of 5% CO_2_ and 95% O_2_, with pH maintained at 7.4.

### Electrophysiological Recordings

Two pairs of silver chloride (AgCl) electrodes placed in agar bridges, made using Krebs without glucose, were used for the Ussing chamber recordings (Figs. 1A, 1B). One pair of electrodes was used to measure the TEP across the tissue. The second pair of electrodes was used to pass a current to clamp the TEP at zero millivolts (Fig. 1B). A DVC-1000 Voltage/Current Clamp unit (World Precision Instruments, Sarasota, FL, USA) (Fig. 1A) was used for voltage clamping and measurements of the resulting I_SC_. The DVC-1000 unit was programmed to pass a current through the tissue, causing a 1-mV deflection in the TEP every 5th minute, and the resulting deflections in TEP and I_SC_ were used to calculate TER, according to Ohm’s law. The TEP was measured in millivolts (mV), the I_SC_ in µA/cm^2^, and TER in ohm·cm^2^. The output from the DVC-1000 unit was digitized by an analog/digital converter unit (PowerLab 16/30; ADInstruments, Dunedin, New Zealand) (Fig. 1A), and the data were collected by LabChart 7 data acquisition software (ADInstruments).

### Experimental Protocols

Both eyes of each animal were used, but only one eye from each animal was used for each experimental series. The number of mice tested in each experimental series was six to eight. Each control period or treatment period lasted 30 minutes. The recording of the I_SC_ was continuous, but for data analysis we took an average of a 10-second period just before each current pulse (and an average of the I_sc_ during the pulse itself), which provided six data points for each 30-minute period to use for statistical analysis and to plot as means in figures.

**Figure 2.**
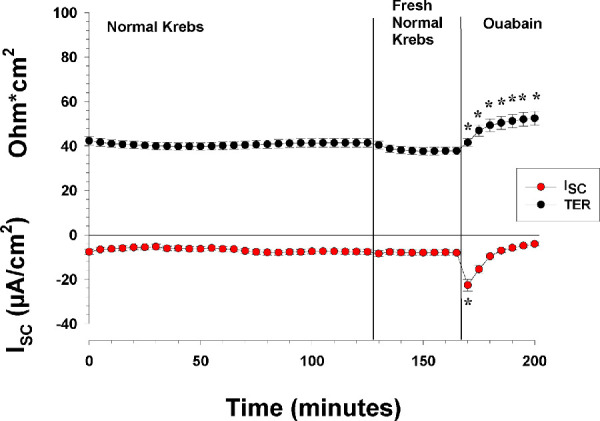
The effects of apical and basolateral normal Krebs and ouabain on the I_SC_ and TER. The figure shows the I_SC_ and TER of control RPE tissue preparations with normal Krebs for 165 minutes. At the 130th minute, the Krebs solution was exchanged with fresh Krebs solution. At the end of the experiment, the Na/K-ATPase blocker ouabain was added to the apical side (n = 6). The measurements with the tissue in normal Krebs solution for 165 minutes indicate that it was stable. When ouabain was added to the apical bath solution, the I_SC_ and TER both changed significantly. ^*^Statistically significant change in the measured I_SC_ and TER.

**Figure 3.**
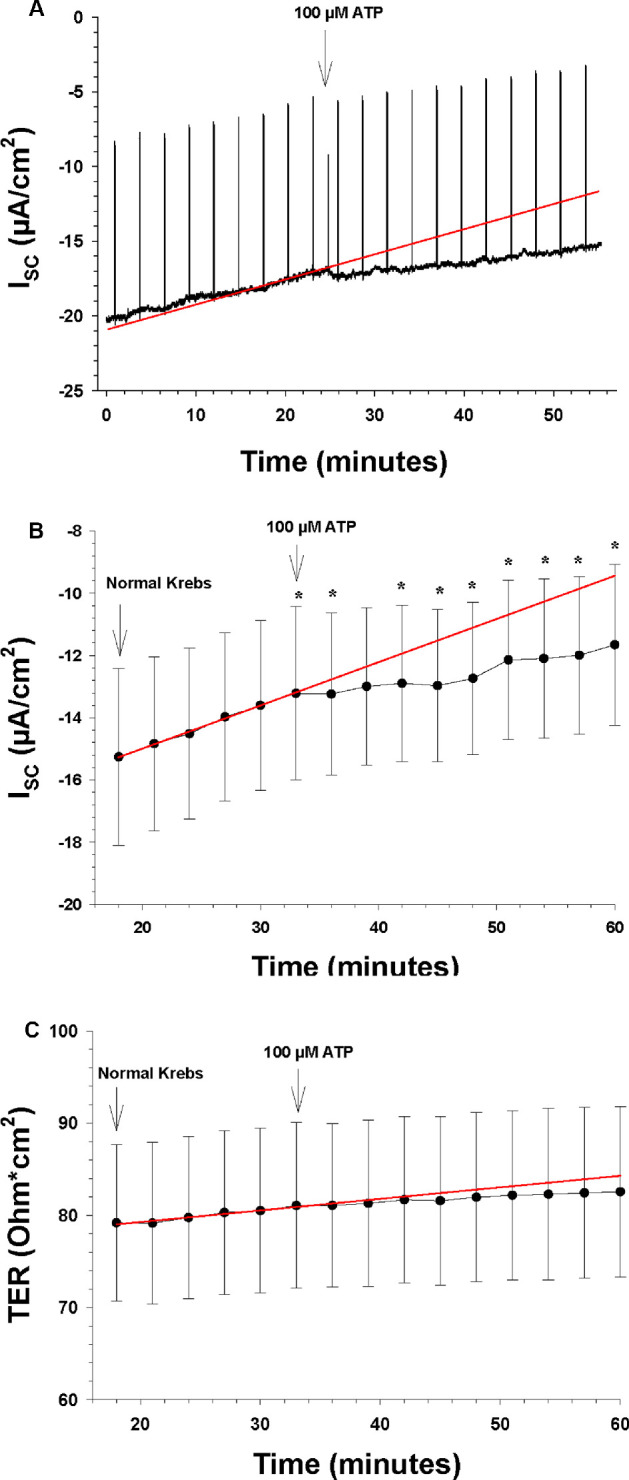
The effects of apical and basolateral ATP on the I_SC_ and TER (n = 7). The *arrows* indicate the time points when a new bath solution was applied to the bath or when the agonist ATP was added to the bath solution. (**A**) Continuous recording of the I_SC_ as a function of time before and after apical and basolateral application of ATP (100 µM). The upward deflections are current responses to voltage pulses of 1 mV every 5 minutes, 1 ms in duration, to measure TER. The *red line* is the control regression line calculated between the last three I_SC_ measurements of the control period at the beginning. The asterisks here and in subsequent figures indicate a statistically significant difference of the measured I_SC_ compared to the control regression line of each time by paired *t*-tests. (**B**) The I_SC_ as a function of time before and after apical and basolateral application of ATP (100 µM). The ordinate shows the measured value of the I_SC_ (in µA/cm^2^), and the *red line* is the control regression line, as in **A**. (**C**) TER as a function of time before and after apical and basolateral application of ATP (100 µM). The ordinate shows the measured value of TER (in ohm·cm^2^), and the *red line* is the control regression line, as in **A**.

**Figure 4.**
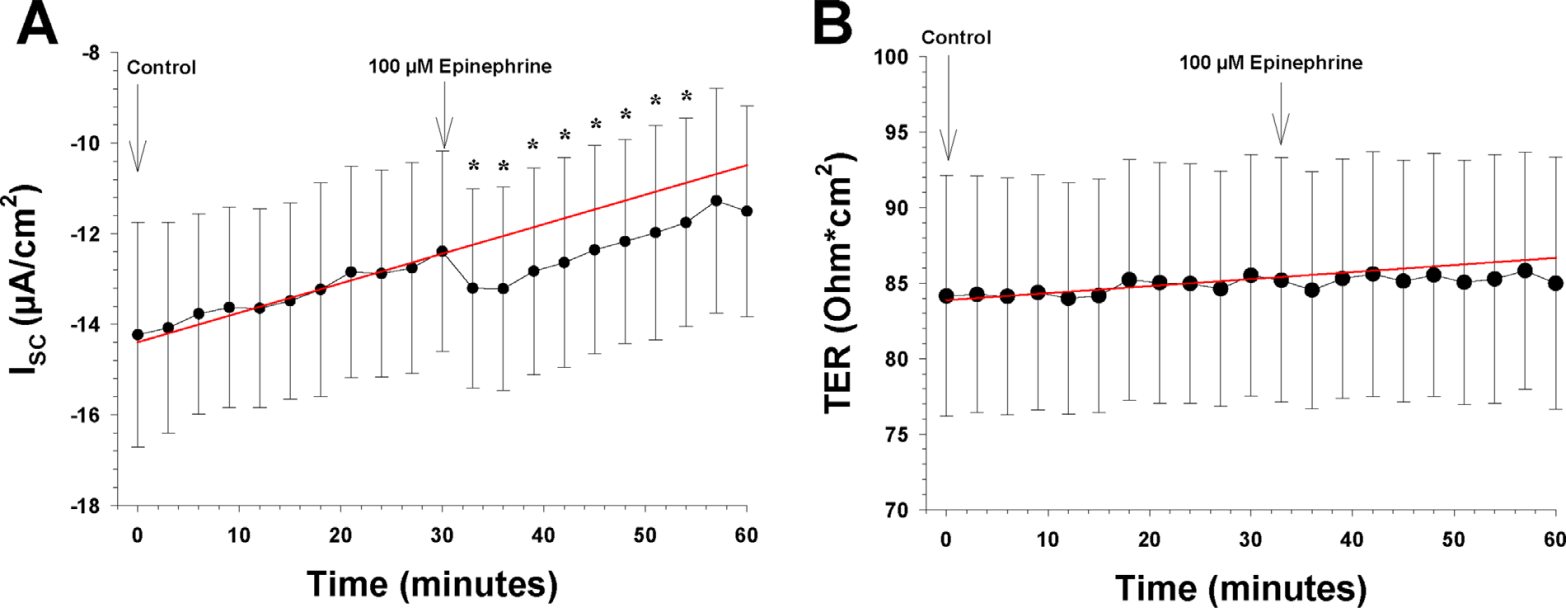
The effects of the apical and basolateral epinephrine on the I_SC_ and TER (n = 7). The *arrows* show the time points when a new bath solution was applied to the bath or when the agonist epinephrine was added to the bath solution. (**A**) The I_SC_ as a function of time before and after apical and basolateral application of epinephrine (100 µM). The ordinate shows the measured value of the I_SC_ (in µA/cm^2^), and the *red line* is the control regression line calculated between the last three I_SC_ measurements of the control period at the beginning. (**B**) TER as a function of time before and after apical and basolateral application of epinephrine (100 µM). The ordinate shows the measured value of TER (in ohm·cm^2^), and the *red line* is the control regression line, as in **A**.

**Figure 5.**
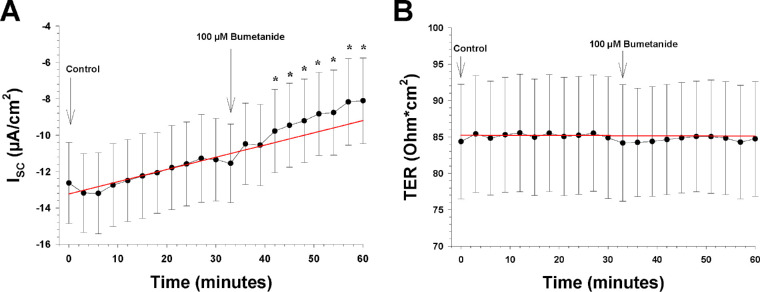
The effects of an apical and basolateral Na-K-2Cl cotransporter blocker on the I_SC_ and TER (n = 7). The *arrows* show the time points when a new bath solution was applied to the bath or when the Na-K-2Cl cotransporter blocker bumetanide was added to the bath solution. (**A**) The I_SC_ as a function of time before and after apical and basolateral application of bumetanide (100 µM). The ordinate shows the measured value of the I_SC_ (in µA/cm^2^), and the *red line* is the control regression line calculated between the last three I_SC_ measurements of the control period at the beginning. (**B**) TER as a function of time before and after apical and basolateral application of bumetanide (100 µM). The bumetanide shows the measured value of TER (in ohm·cm^2^), and the *red line* is the control regression line, as in **A**.

**Figure 6.**
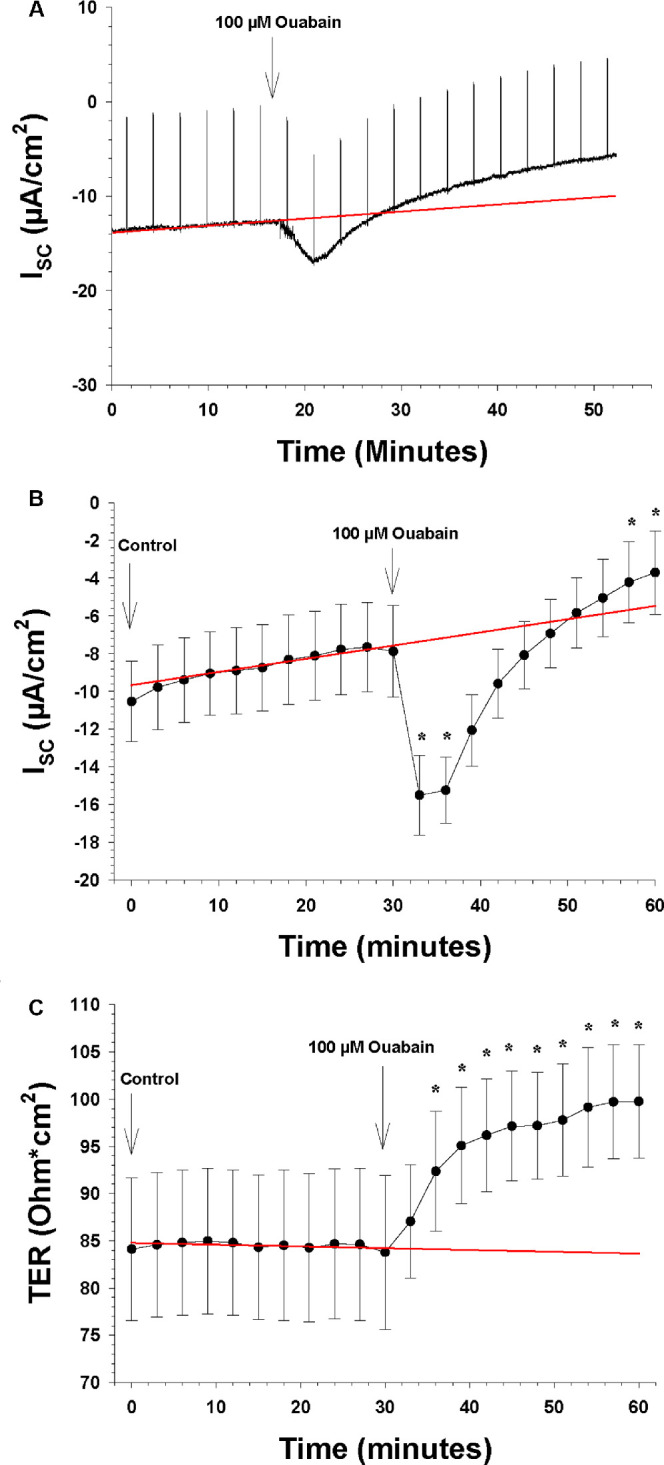
The effects of the apical and basolateral Na-K-ATPase blocker ouabain on I_SC_ and TER (n = 7). The *arrows* show the time points when a new bath solution was applied to the bath or when the Na-K-ATPase blocker was added to the bath solution. (**A**) Continuous recording of the I_SC_ as a function of time before and after the apical and basolateral change of normal Krebs solution to low K^+^ Krebs solution. The upward deflections are current responses to voltage pulses of 1 mV every 5 minute, 1 ms in duration, to measure TER. The *red line* is the control regression line calculated between the last three I_SC_ measurements of the control period at the beginning and the last three I_SC_ measurements of the control period at the end of the experiment. (**B**) The I_SC_ as a function of time before and after the apical and basolateral application of the K-ATPase blocker to the bath solution. The ordinate shows the measured value of the I_SC_ (in µA/cm^2^), and the *red line* is the control regression line, as in **A**. (**C**) TER as a function of time before and after the apical and basolateral application of the Na-K-ATPase blocker to the bath solution. The ordinate shows the measured value of TER (in ohm·cm^2^), and the *red line* is the control regression line, as in **A**.

**Figure 7.**
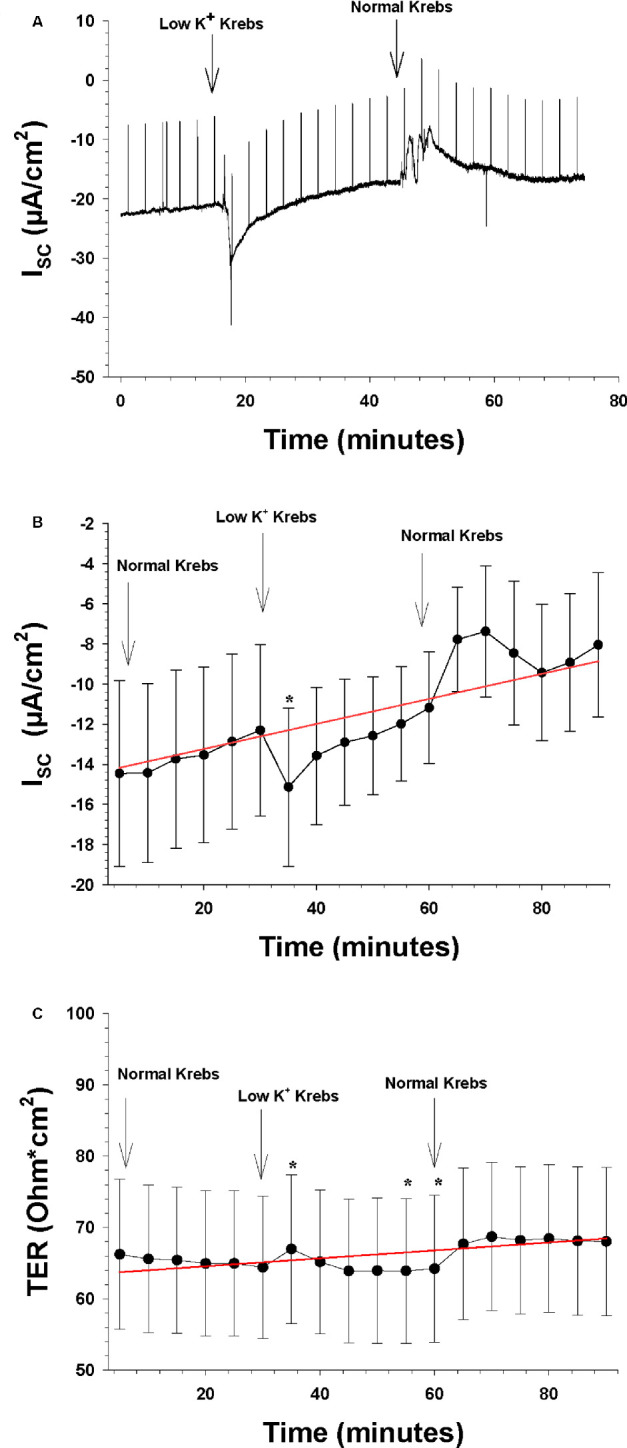
The effects of decreasing apical extracellular K^+^ from 5 to 0 mM (n = 8). The *arrows* indicate the time points when a new bath solution was applied to the bath or when the agonist ATP was added to the bath solution. (**A**) Continuous recording of the I_SC_ as a function of time before and after the apical and basolateral change of normal Krebs solution to low K^+^ Krebs solution. The upward deflections are current responses to voltage pulses of 1 mV, 1 ms in duration, to measure TER. The *red line* is the control regression line calculated between the last three I_SC_ measurements of the control period at the beginning and the last three I_SC_ measurements of the control period at the end of the experiment. (**B**) The mean I_SC_ as a function of time before and after the apical and basolateral change of normal Krebs solution to low K^+^ Krebs solution. The ordinate shows the measured value of the I_SC_ (in µA/cm^2^), and the *red line* is the control regression line, as in **A**. (**C**) TER as a function of time before and after the apical and basolateral change of normal Krebs solution to low K^+^ Krebs solution. The ordinate shows the measured value of TER (in ohm·cm^2^), and the *red line* is the control regression line, as in **A**.

**Figure 8.**
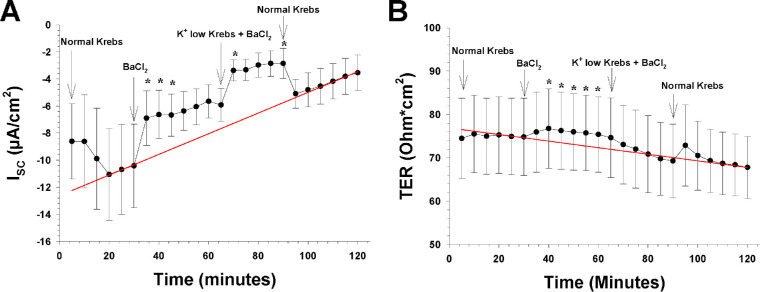
The effects of blocking barium-sensitive K^+^ channels on both sides by adding 1-mM BaCl_2_ to the extracellular fluid (n = 8). The *arrows* show the time point where a new bath solution was applied to the bath or when the barium-sensitive K^+^ channel blocker BaCl_2_ was added to the bath solution. (**A**) The I_SC_ as a function of time before and after the apical and basolateral application of BaCl_2_ (1 mM) and 30 minutes later the change to low K^+^ Krebs solution (0-mM K^+^ concentration) with BaCl_2_ (1 mM). The ordinate shows the measured value of the I_SC_ (in µA/cm^2^), and the *red line* is the control regression line calculated between the last three I_SC_ measurements of the control period at the beginning and the last three I_SC_ measurements of the control period at the end of the experiment. (**B**) TER as a function of time before and after the apical and basolateral application of BaCl_2_ and the change of normal Krebs solution to low K^+^ Krebs solution with BaCl_2_ (1 mM). The ordinate shows the measured value of TER (in ohm·cm^2^), and the *red line* is the control regression line, as in **A**.

In all experiments, the tissue was allowed to stabilize in the Ussing chamber for at least 30 minutes, after which a control period (Control 1) was recorded for 30 minutes, except in the first experiment depicted in Figure 2, where the initial control period lasted 2 hours. In the second set of experiments, there was only one initial control period followed by 30-minute drug periods (Figs. 3–6). In the later ion exchange and barium experiments, a second control period was added at the end, where the depleted ions were replenished again and the drugs were washed out (Figs. 7–8).

In the first experiment depicted in Figure 2, which was to test the viability of the retina/RPE/choroid/sclera preparation under the normal conditions of the Ussing chamber environment, the preparation was left without any experimental manipulation for 2 hours, after which the Krebs solution was replaced with a fresh solution; finally, 1-mM ouabain was added to the apical solution. In the second set of experiments, after the initial control period, the following substances were added in the order listed: ATP (100 µM), epinephrine (100 µM), bumetanide (100) µM), and ouabain (1 mM). All of the experimental substances were added to both the apical and basolateral side of the tissue. In the ion exchange and BaCl_2_ experiments, after the initial control period (Control 1), the normal Krebs solution was washed off three times with K^+^-free Krebs. After 30 minutes of recording, these solutions were replaced three times with fresh normal Krebs, and the I_SC_ and TER were recorded for another 30 minutes (Control 2). After Control 2, we examined the effect of adding the potassium channel blocker BaCl_2_ to both sides with the tissue bathed in the normal Krebs, after which we changed to K^+^-free Krebs, followed by a Control 3 period with normal Krebs only. In the data analysis, we treated these treatments separately for the sake of clarity, such that the first ion exchange experiments without BaCl_2_ were treated as one experimental series, and the second ion exchange experiments with BaCl_2_ were treated as another experimental series. Thus, the Control 2 period of the ion exchange experiments without BaCl_2_ is the same period as the Control 1 period of the ion exchange experiments with BaCl_2_.

It was found that the absolute values of the I_SC_ were variable, resulting in large deviations of the means; therefore, all of our tests of significance are based on paired *t*-tests. We compared the measured values of I_SC_ and TER at each time point of the experimental period to regressed values at the same time point calculated from regression lines derived from the previous period, or between two control periods (see Data Analysis), rather than, for example, comparing the last time point of the previous period with the last time point of the experimental period, which would have led to erroneous conclusions due to the time factor involved. The regression method coupled with the paired *t*-test is, in our opinion, a simple but powerful method to eliminate both the time factor and the variability of the absolute I_SC_ and TER values, particularly when using two control periods, one before and one after the experimental periods, as done in the later experiments presented here. Thus, all statistical *P* values hereafter refer only to comparisons between the measured I_sc_ and TER values and the corresponding extrapolated control values along the control regression line at the same time points.

### Data Analysis

In preliminary experiments, it was observed that the I_SC_ and TER of the RPE preparation generally changed slowly with time, which is a common problem encountered with measurements of epithelial tissue in Ussing chambers, possibly due to degeneration of the cells in the tissue. To control for the time factor, the effects of each treatment (drug or ion exchange) were evaluated by comparing the measured I_SC_ and TER values with calculated I_SC_ and TER values at the same time points according to a linear regression line extrapolated either from the previous control or experimental period (first and second experiments) or between the Control 1 and Control 2 periods (all other experiments). The values used for the regression line in each case were either all of the points of the previous period or the last three points of the Control 1 period plus the last three points of the Control 2 period. These methods are depicted graphically in Figure 3A. The difference at each time point between the measured I_SC_ and TER and the values calculated from the slope and intercept of the control regression line were tested by the paired option of the Student's *t*-test in Excel 2010 (Microsoft, Inc., Redmond, WA, USA). A value of *P* below 0.05 was considered significant for a treatment. In some cases, the mean ± SEM slopes of the I_SC_ are reported with paired *t*-test comparisons between control and experimental periods. Also, the percentage change in either I_SC_ or TER was calculated in some cases, based on the deviation of the measured values at each time point from the extrapolated values derived from the control regression lines. The figures were made using SigmaPlot Version 13 (Systat Software, Inc., San Jose, CA, USA). All results are reported or depicted as means ± SEM.

## Results

### Baseline Electrophysiological Values

In the series of experiments with only normal Krebs present for 2 hours, n = 6 (Fig. 2), there was no significant change in either the I_SC_ or TER over this period of time. The mean I_SC_ of the last 50 minutes was −7.7 ± 2.7 µA/cm^2^, with the basolateral side being negative, and the mean TER was 41.2 ± 4.8 ohm·cm^2^. These values indicate a low TEP, 0.32 mV (apical side positive), based on Ohm's law. After a 2-hour bath, the normal Krebs solution was replaced with a fresh normal Krebs solution, which caused no change in the I_SC_ and only a small decrease in TER (about 3 ohm·cm^2^). In most, if not all, of the species studied an active Na/K-ATPase-dependent mechanism has been demonstrated on the apical side of the retinal pigment epithelium, which can be effectively blocked by ouabain.^13^ We therefore tested the viability of the RPE preparation by adding ouabain to the apical side. This resulted in a rapid and large increase in the negative I_SC_, which nearly tripled from −8.14 ± 2.96 to −22.66 ± 6.34 µA/cm^2^ in about 5 minutes, after which the I_SC_ decreased and reached −4.16 ± 2.21 µA/cm^2^ after 30 minutes, which was significantly below the control regression line (*P* = 0.011). Ouabain caused TER to increase steadily, and after 30 minutes it had increased significantly to 52.46 ± 7.70 ohms·cm^2^ (*P* = 0.007). These results indicate that the RPE preparation was in good condition for 165 minutes and responsive during that time to drugs, as demonstrated by the ouabain response (Fig. 2).

We also calculated the means of I_SC_ and TER of the Control 1 periods of all of the mouse preparations that were tested with drugs or ion exchange, and these values are shown in Table 1. The mean I_SC_ ranged between 4 and 18 µA/cm^2^, and the mean TER ranged between 44 and 86 ohm·cm^2^, which resulted in a low calculated TEP in almost all of the experiments, around 1 mV (Table 1).

**Table 1.** Mean Values of the Electrophysiological Parameters at Initial Baseline

Parameter	Mean	SEM	*n*	Unit
I_SC_	–11.95	3.83	23	µA/cm^2^
TER	67.06	7.99	23	ohm·cm^2^
TEP	0.98	0.34	23	mV

Shows the means and SEMs of the recorded I_SC_ and TER, except for the mice in the control series depicted in Figure 1. The values were taken at the end (30th minute) of the Control 1 period. The TEP was calculated by Ohm's law from the I_SC_ and TER for each animal, before calculating the reported mean and SEM. The total number of experiments performed is indicated.

We found that the stable I_SC_ measured in the control series shown in Figure 2 was the exception rather than the rule, as it was more common that the I_SC_ decreased slowly with time, as evident from the experimental series shown in Figures 3 to 8. This has previously been observed in bovine RPE tissue.^25^ In our case, the decrease in I_SC_ was 0.078 ± 0.033 µA/cm^2^·hr^−1^ with an intercept at −14.0 ± 4.1 µA/cm^2^ (mean slopes of all Control 1 periods). This phenomenon was taken into account in our data analysis (see Methods). Although TER was found to be generally steady during the control periods of most experiments, we analyzed the TER data in a similar way.

### Effects of Purinergic and Adrenergic Agonists, and Transporter Blockers

In the second series of experiments (n = 7), we tested four drugs in an additive manner, with only one control period at the beginning. Each drug tested was applied simultaneously on both the apical and basolateral sides. The results are here depicted for each substance in Figures 3 to 6, where the control values for each substance were obtained during the 30-minute period immediately before the addition of that substance. As Figure 3 shows, there was a steady but slow decrease in the negative I_SC_ during the control period, and this decrease seemed to continue during the entire course of these experiments, independent of the effects of drug applications.

The first drug tested was ATP (100 µM). As can be seen from Figure 3B, the slope of the negative I_SC_ decreased significantly from 0.097 ± 0.046 to 0.041 ± 0.028 µA/cm^2^/min (*P* = 0.040), indicating that the ATP induced an increase in the I_SC_, which counteracted the underlying decrease in the I_SC_. When the measured I_SC_ values were compared by paired *t*-test to the regressed values at the same time points, the difference between these values was highly significant within 6 minutes (*P* = 0.018), and this difference increased as time progressed, reaching 2.22 ± 0.41 µA/cm^2^ after 30 minutes (*P* = 0.003). This amounts to a 24% increase. ATP had no effect on TER (Fig. 3C).

We then added 100-µM epinephrine to both sides of the tissue with ATP still present, as shown in Figure 4A, and the tissue was left in the bath for an additional 30 minutes. Epinephrine caused a small but rapid increase in the I_SC_ of 1.14 ± 0.19 µA/cm^2^ (*P* = 0.011), which lasted for 30 minutes. No further change occurred, as evidenced by no difference in the slope of the I_SC_ over time, which remained at 0.046 ± 0.031 µA/cm^2^/min. There was no significant change in TER during epinephrine application (Fig. 4B).

Bumetanide (100 µM) was then added with both ATP and epinephrine present in the Ussing chambers to block the Na-K-2Cl transporter.^14^^,^^26^ It induced a reduction in the I_SC_, which reached significance after 12 minutes (*P* = 0.009), with a decrease of 1.72 ± 0.73 µA/cm^2^ after 30 minutes (*P* = 0.027). The slope increased from 0.052 ± 0.015 to 0.130 ± 0.024 µA/cm^−2^/min (*P* = 0.022). The blocker did not induce any significant changes in TER (Fig. 5).

Finally, ouabain (1 mM) was added to the Ussing chambers and to both sides, as with the previous drugs. As Figure 6B shows, there was a biphasic response as observed in a previous experiment (Fig. 2), with I_SC_ increasing rapidly in 6 minutes from −7.9 ± 2.4 to −15.49 ± 2.12 µA/cm^2^, which is an increase of 6.92 ± 2.76 µA/cm^2^ (*P* = 0.023). A gradual decrease followed thereafter and reached −3.7 ± 2.2 (*P* = 0.031) after 25 minutes. Ouabain also induced an increase in mean TER, from 83.8 ± 8.2 to 99.8 ± 6.0 ohm·cm^2^ in 30 minutes (*P* = 0.017) (Fig. 6C).

### Role of Potassium Channels

To mimic the light-induced changes in the subretinal space, which has been found to induce a decrease in subretinal [K^+^]_o_ from 5 to 2 mM,^3^ we reduced the apical [K^+^]_o_ to 0 mM. The results are shown in Figure 7B (n = 8). This caused a transient significant increase in the negative I_SC_ during the first 5 minutes by 3.36 ± 1.06 µA/cm^2^ (i.e., from −14.15 ± 4.37 to −17.43 ± 3.65 µA/cm^2^; *P* = 0.008). This increase remained steady during a significant portion of the 30-minute period when the zero-K^+^ Krebs solution was present in the bath. Returning the apical [K^+^]_o_ to normal levels evoked a change in the opposite direction, as the I_SC_ decreased by 3.45 ± 1.60 µA/cm^2^ at peak (*P* = 0.034), which then returned slowly to baseline in 20 minutes. TER, which was 70.6 ± 8.9 ohm·cm^2^ immediately before the change, first increased and then decreased significantly compared to the regression line (*P* = 0.02), but the changes were relatively small, approximately 2 ohm·cm^2^ in either direction (Fig. 7C).

We next examined the role of K^+^ channels in the net transepithelial ion transport across the RPE preparation. After the RPE tissues had adjusted to the normal Krebs solution again for 30 minutes, we blocked barium-sensitive K^+^ channels on both sides of the preparation by adding 1-mM BaCl_2_ to the extracellular fluid. The I_SC_ decreased from −10.4 ± 3.1 to −6.6 ± 1.8 µA/cm^2^ (*P* = 0.01), and TER increased from 74.8 ± 9.6 to 75.4 ± 8.7 in 30 minutes, a significant change compared to the regression line (*P* = 0.03) (Fig. 8). After a 30-minute period when barium was present in the bath, the normal Krebs solution was replaced with a 0-mM K^+^ Krebs solution, keeping the barium concentration at 1 mM, to test for the role of barium-sensitive potassium channels in the observed response to apical K^+^-free solution. When analyzing this part of the experiment, the barium period was used as Control 1. The I_SC_ decreased significantly from −5.65 ± 1.24 to −3.37 ± 0.79 µA/cm^2^ (*P* = 0.029) 10 minutes after switching to apical K^+^-free solution with barium, and it remained significantly different from the control regression line for the next 20 minutes (Fig. 8A). TER decreased significantly and returned to the pre-barium level, as indicated by the regression line between the two control periods in Figure 8B.

## Discussion

The electrical correlates of ion transport across the RPE have been recorded from a number of vertebrate species, although the mouse RPE has been a notable exception so far. Here, we establish that, despite the small size of the mouse eye, the I_SC_ and TER of the RPE can be reliably recorded from a mouse retina/RPE/choroid/sclera preparation in the modified Ussing chamber system with an aperture of 0.031 cm^2^ as used in this study. Both the I_SC_ and TER recorded from the preparation were stable over more than 2 hours. The effects of a Na-K-ATPase blocker, ouabain, demonstrated that the RPE preparation was live and responsive after this long time. A slow, gradual reduction of the I_SC_ occurred over time, comparable to what has been observed in long-term recordings from similar preparations.^4^^,^^25^^,^^26^ We took this natural decrease of the current into account in all our analyses.

The present study, which, to our knowledge is the first to examine ion transport across the mouse RPE, has several limitations. The mean values of the I_SC_ and TER are presented in Table 1. These values are rather low compared to preparations from other species^1^ and resulted in a low calculated TEP, around 1 mV, with the apical side being positive, indicative of a rather leaky preparation. Nevertheless, we found the preparation to be robust and responsive to various treatments that affect ion transport, lasting for 2 to 3 hours. One difference between the mouse preparation used in the present study and larger ones from other species used in previous work is that both the neuroretina on the apical side and choroid and sclera on the basolateral side were kept in situ. This may constitute a diffusion barrier to drugs reaching the RPE, and thus represents a limitation of our study, but the present results suggest that is not a serious hindrance to the use of this preparation, as most responses were rapid and within seconds. Because the preparation leaves the retina in situ, Müller cells are still present; spatial buffering of K^+^ from the RPE to the vitreous side mediated by these glial cells is likely to occur and may have affected measurements of ion transport, which can be considered a limitation of the study.

TER values presented in this study are calculated as ohms·cm^2^ and are based on passing a current very briefly through the tissue every 5th minute during the experiments, causing a 1-mV deflection of the TEP from the clamped value of 0 mV. The corresponding shift in the I_sc_ required to keep the TEP at 1 mV was then measured, and these deflections in the TEP and I_sc_ were then used to calculate TER based on Ohm's law. The TEP, TER, and I_sc_ values thus obtained can only be regarded as an indication of the non-clamped or open circuit values, as the ion transport capabilities and ion gradients across the tissue under voltage-clamped conditions are not the exact same as under an open circuit. The above calculations are also based on the assumption that there was no unnatural leakage through the preparation, which is probably not true but difficult to ascertain. We suspect that some leakage is the reason for the relatively low TEP values found and was perhaps caused by edge damage that may have occurred during mounting of the tissue in the Ussing chambers or was due to discreet damage caused by preparation of the tissue that was not visible in the dissecting microscope. The TER values calculated are based on the assumption that the surface area of the tissue was nearly the same as that of the opening area of the Ussing chamber, which was 0.0013 cm^2^. However, it is likely that the area of the RPE is actually larger than the opening, given that there are basal deep infoldings and apical microvilli that increase the surface area of the RPE and probably not to the same extent on the apical and basolateral surfaces. The TER values should thus be regarded as a close approximation of the actual transepithelial resistance in our preparation.

Table 2 summarizes the effect of the various treatments known to affect RPE ion transport that were examined in this study, in comparison with similar findings from other mammalian species. It should be noted that some of the studies referred to in Table 2 combined recordings from the RPE in a classic Ussing chamber preparation similar to the one used here with intracellular recordings from individual RPE cells.^6^^,^^10^^,^^25^^,^^27^^–^^30^ ATP has been shown to increase transepithelial ion and fluid transport across the RPE measured in Ussing chambers^29^^–^^31^ and to induce ionic currents in isolated rat RPE cells studied with the patch clamp technique.^32^ ATP is converted into adenosine monophosphate (AMP) by the eNPP family of enzymes, and AMP then dephosphorylates to adenosine by ecto-5′-nucleotidase, and this process has been shown to be present on the apical side of the RPE.^33^ This conversion of ATP is rapid; therefore, it cannot be ruled out that the effects of ATP were in part mediated by adenosine receptors, as well as P2 purine receptors. Both P2X and P2Y receptors, as well as adenosine receptors, have been found on RPE tissues in various animals. But, up to this point, it is not known which subtypes of P2Y receptors are expressed in the mouse RPE, and an extensive literature search suggests that this has not been examined.

**Table 2.** RPE Responses of Four Mammalian Species to the Substances Tested

	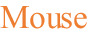		Human		Bovine		Rabbit		
	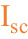	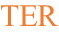	I_sc_	TER	I_sc_	TER	I_sc_	TER	References
ATP	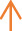	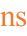	↑	↓	↑	↑	NT	NT	^29^ ^–^ ^31^
Epinephrine	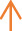	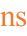	↑	↓	↑	ns	↓	ns	^9^ ^–^ ^11^ ^,^ ^25^ ^,^ ^27^
Bumetanide	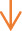	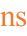	↓	ns	↓	↓	ns	ns	^6^ ^,^ ^9^ ^,^ ^15^ ^,^ ^26^
Ouabain	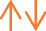	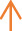	↑↓	ns	↑↓	ns	↓	ns	^4^ ^,^ ^10^ ^,^ ^25^ ^,^ ^39^ ^,^ ^42^
Low apical K^+^	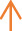	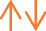	↑	↑	↑	↓	NT	NT	^10^ ^,^ ^15^ ^,^ ^25^ ^,^ ^31^ ^,^ ^46^
Ba^++^	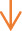	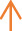	↓	↑	↓	ns	NT	NT	^6^ ^,^ ^10^ ^,^ ^15^ ^,^ ^25^ ^,^ ^46^ ^,^ ^47^

NT = not tested/no data available; ns = no significant effect.

Summary of the results from the present study in comparison with findings from three other mammalian species. The arrows indicate the effect—increase (upward) or decrease (downward)—of a drug or substance on I_sc_ and TER of the RPE in our experiments with mouse RPE (colored arrows). The black arrows represent the effects of a drug or substance on I_SC_ and TER found in previous studies on RPE in three other mammalian species.

It has been shown that in both the bovine and human retinal pigment epithelia, epinephrine stimulates transport of fluid and ions such as Cl^–^ and K^+^ across the RPE via alpha-1 receptors located on the apical side.^10^^,^^11^^,^^27^^,^^34^ It is believed to act in vivo as a paracrine signal from the retina to the RPE.^9^^,^^11^ It has been known for some time that adrenergic receptors are present on RPE cells,^35^ and recently it has been shown that there are adrenergic nerve endings in close proximity to the basolateral membrane of the mouse RPE and β1 and β2 adrenergic receptors are present on mouse RPE cells.^36^ Unlike what has been found in rabbit,^34^ bovine,^9^ and human RPE,^10^ the effect of epinephrine on the mouse RPE short-circuit current was small, but significant, whereas there was no effect on TER. This is similar to what has been found in the rabbit RPE^34^ (Table 2). Epinephrine also does not affect TER measured from the bovine RPE/choroid,^9^ but it lowers TER measured from a human fetal RPE/choroid preparation.^10^^,^^11^ Based on these findings, our results suggest that epinephrine has a limited function as a paracrine signal affecting ion transport and fluid absorption in the mouse RPE.

The effect of epinephrine on the RPE transport has been found in some species to be dependent on the apical Na-K-2Cl cotransporter,^9^^,^^10^ as bumetanide blocks the adrenergic effect on the apical side of the RPE (Table 2). This includes blockage of the effect of epinephrine on fluid transport across the bovine RPE.^9^ The effects of blocking the Na-K-2Cl cotransporter of the mouse RPE with bumetanide are comparable to its effects on the short-circuit current found in tissue from several other species,^6^^,^^14^^,^^28^ As found in the mouse, bumetanide induces little or no change in pigment epithelial total resistance in any of the species tested. The apical Na-K-2Cl cotransporter on the RPE serves a vital role in the transport of ions and fluid across the tissue. Under normal conditions, the bovine RPE has been found to absorb chloride across the apical membrane, and this absorption is blocked by apical bumetanide.^15^ The cotransporter also has been found to absorb K^+^ across the apical membrane of the bovine RPE.^15^ Thus, the role of the Na-K-2Cl cotransporter in the RPE seems to vary between species (Table 2), and the present results suggest it plays a lesser role in the function of the mouse RPE than in most of the other species examined, except the rabbit. It should be noted, however, that the Na-K-2Cl cotransporter, as well as other RPE ion transport mechanisms, may function differently under an open-circuit condition than the short-circuit condition that was applied in the present work.^1^ This may to some extent account for the variability in the results reported with bumetanide, as some of the previous studies were done under open-circuit conditions while measuring the TEP.

Na/K-ATPase is very important for transepithelial ion transport across the RPE tissue, as it maintains the ionic homeostasis of the RPE cells.^13^^,^^37^^–^^39^ It is electrogenic; for example, it has been shown to create a membrane voltage of about 5 to 10 mV in the bovine RPE.^25^ This electrogenic effect is quickly removed by apically applied ouabain.^37^ In retinal pigment epithelia in general, the Na/K-ATPase is situated on the apical surface.^25^^,^^40^ Blocking the Na/K-ATPase transporter with ouabain applied to either the apical side only or both sides of the mouse retina/RPE/choroid preparation evoked a two-phase response in the I_SC_. These results are comparable to what has been found in most other species when ouabain has been applied to the apical side of the RPE^4^^,^^13^^,^^17^^,^^25^^,^^37^^,^^41^^–^^43^ (Table 2). It has been suggested that the first phase is due to blocking of the electrogenic current generated by the Na/K-ATPase activity, and the second phase is a slow run down of ionic gradients across the apical membrane leading to a gradual depolarization for hours.^37^ Thus, our results with ouabain are consistent with an apical Na/K-ATPase transporter being present on the mouse RPE that shows electrogenic activity. When ouabain is applied to the basolateral side of the RPE, there is no effect on the I_SC_ or the TEP, which is consistent with a distribution of RPE Na/K-ATPase subunits being restricted to the apical membrane surface.^4^^,^^25^^,^^40^ As the experiment shown in Figure 2 demonstrates, these effects of ouabain are due to its blockage of apically located Na/K-ATPase. It should be emphasized that ATP, epinephrine, bumetanide, and ouabain were added to the bath sequentially, which may have to some extent altered the response of the tissue, but the effects of all of these (except for ouabain) on ion transport were reversible, so this approach is not likely to have affected the main conclusions of the study.

Light stimulation induces a decrease in subretinal [K^+^]_o_, caused by the hyperpolarization of the photoreceptors in response to light, which remains lowered to a great extent during maintained light.^44^^,^^45^ It has been suggested that RPE chloride transport can be activated by alterations in K^+^ concentration that occur in the subretinal space, particularly during transitions between light and dark.^15^ The effect of subretinal potassium on RPE transport is dependent on K^+^ channels present on the apical membrane. We found that lowering the apical [K^+^]_o_ produced a pattern in the I_SC_ similar to that observed in the TEP of a bovine RPE/choroid preparation.^25^^,^^46^ Only minor effects on TER were found in these experiments. Although our results generally show a pattern similar to that of the bovine results,^25^^,^^46^ they deviate somewhat, as the I_SC_ did not decrease below previous levels but remained higher during most of the remainder of the experiment (Fig. 7B).

It seems that the transepithelial ion transport of the RPE is dependent on barium-sensitive K^+^ channels, as indicated by the findings summarized in Table 2. We found that 1-mM barium on both sides of the mouse RPE preparation caused a significant decrease in I_SC_ and a small increase in TER. These results are also comparable to changes found in TER measures from several other mammalian preparations (Table 2).^25^^,^^47^ Previous results from the bovine RPE/choroid preparation suggest that a Ba^++^-sensitive apical K^+^ conductance is critical in mediating the RPE response to [K^+^]_o_ changes in the subretinal space.^6^^,^^25^^,^^46^ This was also the case in the present mouse RPE preparation, as barium not only blocked the response to lowering the apical K^+^ concentration but also caused a further decrease in the I_SC_ by approximately 35% (Fig. 8A).

Three main types of K^+^ channels have been found on RPE cells of most species examined in patch-clamp experiments, although the heterogeneity of these channels is considerable: inward rectifier channels blocked by extracellular Ba^++^, outward rectifier voltage-gated K^+^ channels (KCNQ/K channels), and Ca^++^-activated K^+^ channels.^3^^,^^8^^,^^48^^–^^51^ However, whole-cell patch-clamp recordings from cultured mouse RPE cells show that they primarily express a delayed rectifier outward K^+^ current.^52^ Although the effects of Ba^++^ on these K^+^ currents in mouse RPE cells has not been examined, this may to some extent account for the differences in the effect of extracellular Ba^++^ and lowering the apical [K^+^]_o_ on the I_SC_ and TER recorded from the mouse retina/RPE/choroid/sclera preparation as compared to other species tested.^15^^,^^25^^,^^47^^,^^53^ Isolated bovine and human RPE cells show a prominent inward rectifying current, with a conductance that is inversely dependent on the extracellular [K^+^]_o_ and is almost completely blocked by Ba^++^.^8^^,^^49^^,^^54^ Thus, there appears to be a correspondence between the types and relative expression of K^+^ channels on RPE cells of the different species and the effect of extracellular Ba^++^ on the I_SC_ and TER.

Taken together, the present work is, to our knowledge, the first study of ionic transport mechanisms across the mouse retinal pigment epithelium. It allows comparison with similar transport mechanisms in other species, despite differences in size. In addition, a successful and reliable mouse retina/RPE/choroid/sclera preparation can be utilized to examine RPE transport mechanisms in mice with known defects in RPE function. ATP seems to increase transepithelial ion transport across the mouse RPE, and lowering the apical potassium concentration produced results in the I_sc_ similar to those seen previously in TEP. The most salient differences between the present results for mouse RPE and those for other species are that epinephrine appears to have a limited function as a paracrine signal, affecting ion transport and fluid absorption in the mouse RPE. Also, the apical Na-K-2Cl cotransporter may play a lesser role in its function than in most of the other species examined, apart from the rabbit. As found in RPE of other larger mammals, part of the transepithelial ion transport is dependent on potassium channels, which also are part of the response to low subretinal K^+^ postulated to cause the light-induced effects on RPE ion transport. With the use of a modified Ussing chamber, with an aperture as small as 0.031 cm^2^ in diameter, the mechanisms of ionic transport across the normal mouse RPE can be examined.
